# Characterization of paleodrainages in desert regions of Saudi Arabia multisatellite images with field based study

**DOI:** 10.1038/s41598-025-89428-9

**Published:** 2025-02-07

**Authors:** Mashael M. Al Saud

**Affiliations:** King Saud University, Riyadh, Saudi Arabia

**Keywords:** Streams, Porous sediments, Pebbles, Radar images, Saudi Arabia, Geology, Geomorphology, Hydrogeology, Environmental sciences, Hydrology

## Abstract

In Saudi Arabia, a number of linear geomorphological features with uncertain origin have been observed from space, but they do not belong to any existed drainage systems. They are ancient watercourses carried water in the past during the Holocene deluge, and they were affected by global climate change and geological processes turning them into dry and buried channels filled by sediments, and these are described as “Paleodrainages”. This study investigated these features primarily based on the integration of multi-satellite images including SRTM DEM for generating stream networks, ALOS-PALSAR which is capable to penetrate the surficial materials, and ASTER for detecting thermal differentiation in terrain surface. The novelty of this study includes the use of more than one satellite images (optical and microwave) with various spectral and optical characteristics, and this has been supported by field verification to investigate the lithological facies of stuffed materials into the detected paleodrainages, plus the classification performed for the detected paleodrainages and this has never been implemented in previous studies. Hydro-geomorphological-based categorization of these paleodrainages was carried out, indicating the majority of SW-NE trending and the presence of routes, unconsolidated sediments and rocks. From the hydrological point of view, these paleodrainages are potential for groundwater storage; and they can be also suitable sites for artificial groundwater recharge; be-sides they represent routs for saltwater intrusion on-land; and these science-based clues represent supportive element for better water resources management in Saudi Arabia.

## Introduction and concepts

The Arabian Peninsula, including the Kingdom of Saudi Arabia, is one of the most water-scarce regions. The rainfall rate does not exceed 150 mm/year with an average temperature of about 35 °C^1^; and potential evapotranspiration exceeding 2000 mm/year^2^. This has been reflected in the limited renewable water resources, besides the excessive withdrawal from fossil water (i.e., non-renewable) which became the only source of water in Saudi Arabia, notably in the inner regions where the convey of desalinated water is often constrained. In Saudi Arabia, the pumping of groundwater is mainly from the deep aquifers with fossil water, that mostly exceeds 1200 m in depth. For example, the depletion of groundwater has been estimated between − 6.9 × 10^− 2^ and − 8.6 × 10^− 2^ cm/month as detected by GRACE TWS in northern Saudi Arabia, particularly in agricultural areas^3^.

Lately, rainfall patterns in the entire Arabian Peninsula have been changed towards torrential rain revealing an increasing trend in climatic extremes, and evidencing an aspect of climatic variability in the region as it was presumed by^4^. The excessive precipitation occurs in a short time (i.e. torrential rain) has been well pronounced lately and reflected on the increased number of flash floods in many wadis of Saudi Arabia resulting in severe damage to the infrastructure and the environment^5^. Similarly, in many arid and semiarid regions, there are several dams constructed along valleys in Saudi Arabia either to capture water runoff for water supply or to give a chance for the accumulated water behind these dams to infiltrate, as a spontaneous groundwater recharge process^6^.

Even though the Arabian Peninsula, including Saudi Arabia, is characterized by arid climate and scarce water resources, yet this region is well known in the past by numerous surficial watercourses (i.e., rivers and streams) that associated with Mega Aquifer Systems^7–9^. These watercourses were evidenced by^10^ who attributed them to the Holocene deluge; while^11^ stated that these ancient (buried) watercourses were formed during Mid-Late Quaternary (Pleistocene) age, and they were affected later on by paleo-climate and environmental changes. Several studies tackled these hydro-geomorphological features (described as *paleodrainages*), which are buried under desert^12–16^; and some studies were specifically applied for Saudi Arabia^17–20^.

Paleodrainages in Saudi Arabia are many, and they can be observed on satellite images. anomalous drainage systems often characterize the localities where these drainages occur, such as the sharp change in the drainage pathways, compressed meanders, abrupt and localized drainage braiding, and this was described by (Howard, 1967; Al Saud, 2007)^21,22^. Field surveys on the delineated paleodrainages show obvious linear terrain with wet horizons and remarkable vegetation cover among dry sandy lands, also elongated salty soil often occur along these drainages. Based on the hydro-geomorphological concepts, paleodrainages are subsurface conduits characterized by high porosity and permeability and they either store groundwater or transport saltwater to land aquifers.

In many regions, paleodrainages have potential for groundwater storage and they are considered as a solution to address freshwater scarcity notably in arid zones such as in the Eastern Sahara of Egypt and Libya as well as in New South Wales in Australia; and this has been mentioned in many studies^12,23–27^. Besides, the role of paleodrainages in seeping saltwater into the coastal aquifers was also mentioned in may studies^28,29^.

According to Al Saud (2024), the study of paleodrainages in Saudi Arabia is significant because they represent suitable localities for groundwater artificial recharge (GWAR). In addition, Saudi Arabia is frequently witnessing flash floods, and if flood water is harvested and then recharged into these paleodrainages that would, in addition to flood control, feed the groundwater reservoirs, and reduce saltwater intrusions among these channels^5,30^. From the beneficial point of view, paleodrainages are characterized by the following:

1. Highly porous and permeable lithologies (detrital deposits) with considerable hydraulic conductivity.
2. Shallow depth (i.e., few meters) which facilitates groundwater pumping or GWAR mechanism,
3. Low water flow velocity due to the gentle channel slope gradient and this resulted in a minimal water loss,
4. Ease calculation of the dimensions of paleodrainages, especially since their extent and width can be measured by remote sensing tools (i.e., optical and microwave) which can be associated with ground-based measurements.

Usually, paleodrainages are investigated using geophysical techniques where many methods are applied, such as electrical resistivity and conductivity surveys, Ground Propagation Radar (GPR), electromagnetic induction (EMI), Gamma-Spectrometry (GS)^24,31,32^. However, these techniques are time consuming and they are limited by the range of a ground survey, which can affect the extent to which large scale paleodrainages can be surveyed. For this reason, remote sensing, notably the microwave sensor (i.e., radar sensors), became a significant tool for identifying paleodrainages where they are characterized by the capability to penetrate the subsoil and buried surficial materials^33^.

Before the development of remote sensing techniques, the detection of paleodrainages as well as identifying their dimensions, was a challenge; notably they are mostly hidden under detrital, non-consolidated surface materials and loos sand dunes, as well as they are often intermittent^34–36^. Paleodrainages were also subjected to many physical processes (e.g., tectonic activities, climatic events, geomorphological processes, etc.) where their dimensions and flow direction have been changed with time. This has been mentioned in some studies^10,22,37,38^.

The study aims to detect and delineate paleodrainages in a selected desert land from Saudi Arabia where a number of satellite images, with various temporal and spatial resolutions, were processed and then followed by field verification to ensure the presence these buried hydro-geomorphological features. The current study will characterize the identified paleodrainages. The outcome of this study will be a supportive tool that can be help while: (a) assessing potential groundwater storage into these drainages, (b) appraising the possibility of adopting these drainages for groundwater artificial recharge, and (c) identifying the probable interlinkage between saltwater intrusions, several kilometers in land, and the alignment of the identified paleodrainages.

##  Materials and methods

**Fig. 1:**
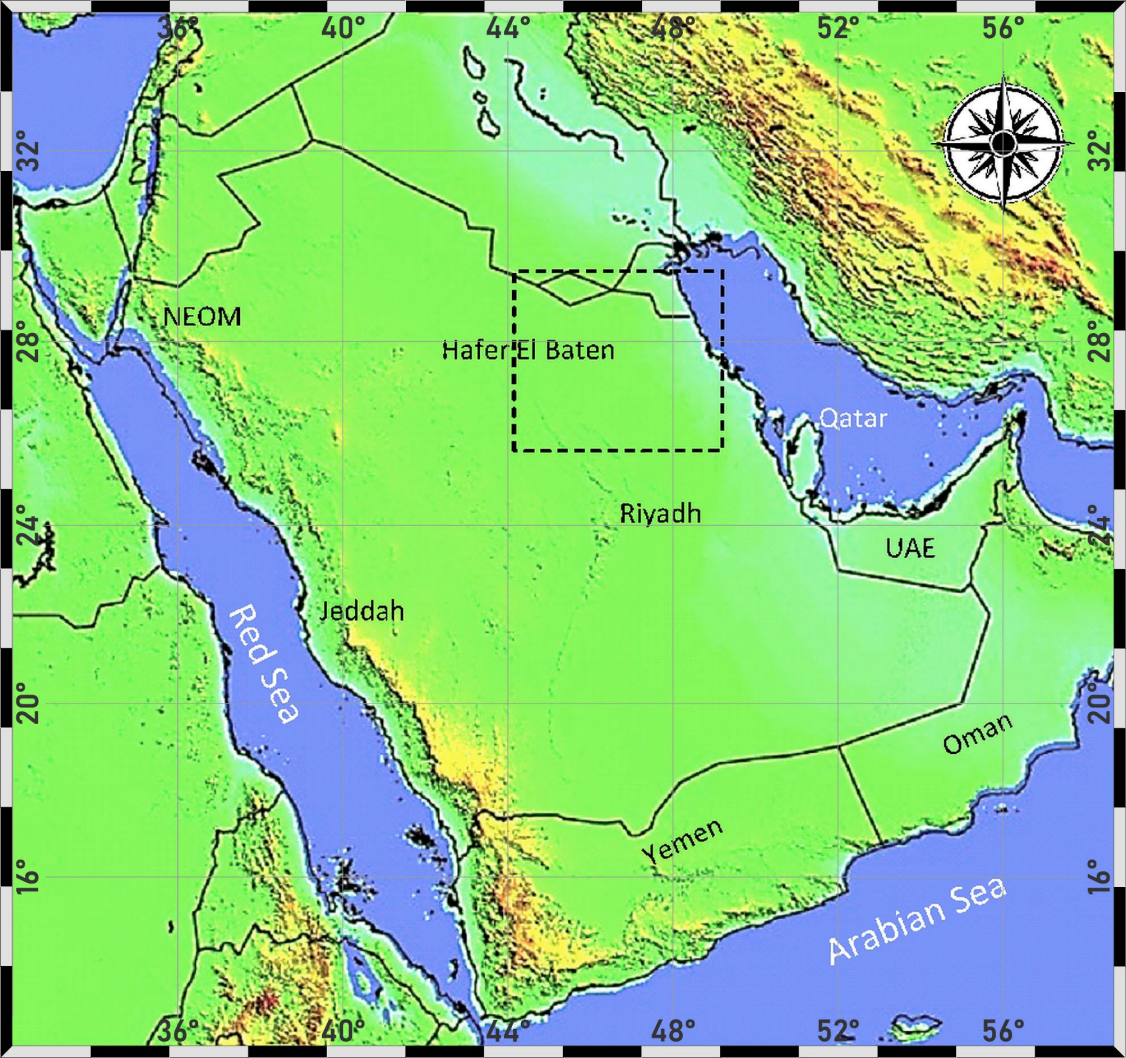
Location map of the study area (frame with dashed line).

The study area, with about 143.000 km^2^, is located in the north-eastern part of Saudi Arabia, between Dammam and Hafer El-Baten to the northern part of Riyadh, which is located between the following geographic coordinates (Fig. 1): 27° 55’ N and 30° 00’ N & 43° 20’ E and 48° 30’ E. The area of study comprises various rock lithologies starting from Triassic Period to Quaternary where thick sequences of carbonates (i.e. limestone and dolomite) and clastic (i.e. sandstone) rocks are interbedded. There is a general sloping trending in the SW-NE direction, and it extends from Al Sawrat Mountain in the west and the escarpment of Tuwaiq in the east^10^. The main surface land is covered by Quaternary deposits and mainly by sandy sediments and desert dunes. In addition, the coastal zone of the study area encompasses large number of Sabkhas (i.e., surficial salt land and lakes) which may extend tens kilometers away from the coast. In this study area, water runoff is mainly diverted from the Arabian Shield (i.e., vast curved mountainous ridges); with dominant slope surface in the NE direction^22^.

### Data sources

To detect paleodrainages and the relevant buried geomorphological features, multi-satellite data was adopted in this study. This included mainly satellite images (optical and microwave remote sensing datasets). Optical remote sensing with multispectral satellite images can detect ground objects with different spectral signatures on satellite images; nevertheless, paleodrainages can be detected using optical sensors with a special emphasis on surficial signatures identified by thermal satellite images where thermal differentiation between surface materials can be discriminated^39,40^. This depends on the fact that paleodrainages are characterized by high porosity and permeability due to the presence of stuffed detrital materials. Thus, paleodrainages represent moist buried conduits that reflecting lower temperature than the surrounding desert lands, and this differentiation in temperature can be detected by thermal satellite images. Moreover, buried channels are a good target for radar notably wetness often causes noise in the signal^24,26^. In addition, the successful detection of paleodrainages can be performed by orbital imaging radar (microwave sensor) which enables the delineation of these ancient hydro-geomorphological features located under terrain surface^12,20,41^.

In this study, three types of satellite images were adopted. These are: (i) Shuttle Radar Topography Mission (SRTM) digital elevation model to delineate drainage systems with multiple dimensions and aspects, (ii) Advanced Land Observing Satellite (ALOS) Phased Array Type L-band Synthetic Aperture Radar (PALSAR) to identify surficial signatures evidencing the delineation of paleodrainages; and (iii) Advanced Space-borne Thermal Emission and Reflection Radiometer (ASTER) to help detecting thermal differentiations in linear moist horizons reflecting buried channels. The retrieved, remote products and their specification are shown in Table 1.

**Table 1:** Remote sensing products and their specifications.

Remote sensing product	Selected dates	Images ID/Granules Path (Long.) & Row (Lat.)	Spatial resolution & bands	Swath width
SRTM DEM	2020	N28, E48 & N27, E46 N28, E47 & N27, E46 N28, E46 & N27, E47 N28, E44 & N27, E48 N28, E43 & N26, E48	-1-arc-second (30 m) -3-arc-second (90 m)	225 km
ALOS-PALSAR	27/2/2020	ALOS2311350550-200227 ALOS2311350540-200227 ALOS2311350530-200227	25 m (L-Band, (1257.5 MHz; λ 25 cm)	70 km
	26/3/2020	ALOS2315490520-200326 ALOS2315490530-200326		
	20-12-2022	ALOS2463200560-221220 ALOS2463200550-221220 ALOS2463200540-221220		
	12-1-2023	ALOS2466600560-230112 ALOS2466600550-230112		
	17-1-2023	ALOS2467340550-230117 ALOS2467340540-230117		
	23-2-2023	ALOS2472810560-230223		
	23/3/2023	ALOS2476950570-230323 ALOS2476950560-230323		
ASTER	08-2019	ASTB190809190913 ASTB190809190922	-15 m (Visible) − 30 m (SWIR) − 90 m (TIR)	60 km
	12-2019	ASTB191219074546		
	03-2020	ASTB200317074033 ASTB200317074024		
	04-2020	ASTB200418074014		
	10-2022	ASTB221004185419 ASTB221004185427

1. Shuttle Radar Topography Mission (SRTM DEM) Digital Elevation Model has been operated by the National Geospatial-Intelligence Agency and NASA for obtaining complete high-resolution topographic datasets for 80% of the Earth’s land surface with data points located every 1-arc-second and 3-arc second with 30 m and 90 m spatial resolution; respectively. SRTM is used with Radar Interferometry where two radar images were retrieved from slightly different locations; and thus, the differences between these images allow the calculation of differentiation in surface elevation. Data was retrieved from: https://www2.jpl.nasa.gov/srtm/.
2. Advanced Land Observing Satellite (ALOS) was launched by the Japanese aerospace Agency JAXA, and it is equipped with three instruments. Among them, the Phased Array Type L-band Synthetic Aperture Radar (PALSAR) which is designed for all-weather Earth observations and to capture images with a spatial resolution of 7 to 100 m. ALOS is occupying a stereo mapping camera. data was retrieved from:

https://www.eorc.jaxa.jp/ALOS/en/palsar_fnf/data/2017/map.htm.

Advanced Space-borne Thermal Emission and Reflection Radiometer (ASTER) is on board NASA’s Terra satellite. It is an advanced multi-spectral sensor ranging from visible to thermal Infrared with 14 spectral bands where 3 of these bands are visible ones and the rest 11 bands are occupied in the infrared range. It is characterized by high spatial resolution of 15 m VNIR, 30 m SWIR and 90 m TIR. Data was retrieved from:

https://gbank.gsj.jp/madas/map/index.html.

In addition to the retrieved satellite images, topographic maps for the study area were used to ensure the delineation of some streams and to compare them with those streams extracted from the SRTM DEM, as well as to identify some domestic names of streams. These maps, which were produced in 1983, are at scale of 1:500.000 and 25 m contour interval as obtained by the Ministry of Petroleum and Mineral Resources^42^.

### Images processing and data analysis

**Fig. 2:**
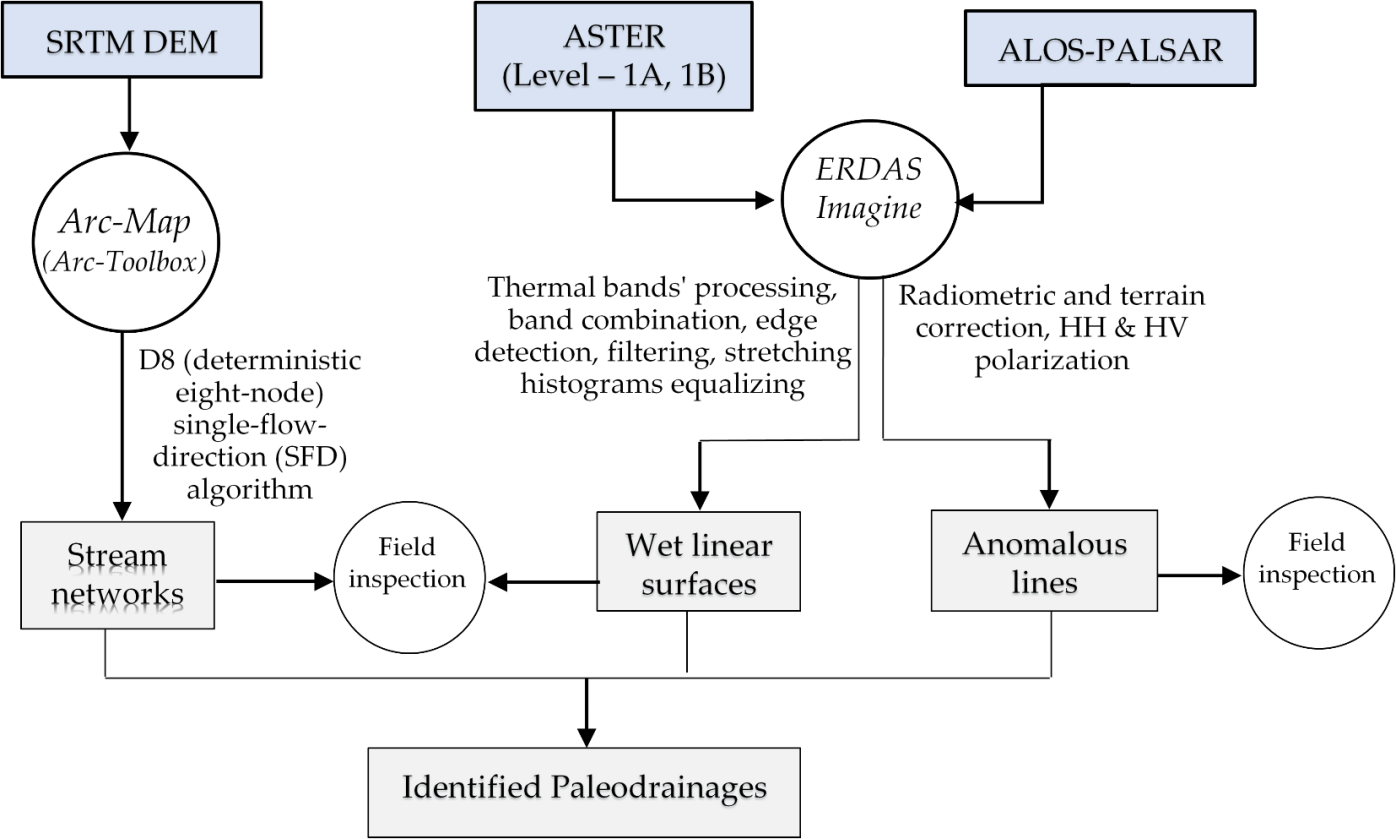
Flowchart showing workflow and the main elements in this study.

Satellite image processing was performed, and their workflow and analysis were illustrated in Fig. 2. SRTM DEM granules for the area of interest (AOI) were downloaded and then they were digitally processed to extract the existing (current) drainage system which can be elaborated to perform different stream orders and dimensions (e.g., reaches, and tributaries) and to identify streams` characteristics including orientation, continuity and the localities where they terminate or disappear.

For this purpose, D8 (deterministic eight-node) single-flow-direction (SFD) algorithm was used to delineate flow from each grid cell to one of eight nearest neighboring ones based on slope gradient which was adopted by O’Callaghan and Mark (1984)^43^. The aspect can be extracted (in degrees clockwise from north) for steepest descent of each grid cell, and also the flow direction from that grid cell. The raster data of SRTM DEM was processed using Arc-Map 10.8, and more certainly in the Arc-Toolbox which is an interface for accessing data conversion and analysis geospatial function. Hence, the “Hydrology” option was elaborated in the Spatial Analyst extension where from fill-in, pit removal–depression filling to filter the digital elevation, and finding outlet cells, were carried out. Therefore, flow direction and accumulation, stream order and stream to feature were determined.

ASTER satellite images have a wide spectral range where its sensor covers three telescopes, and thus it represented a variety of bands as mentioned in Table 1. There are two levels for ASTER satellite images (i.e., Level-1 A and Level-1B) where Level-1 A data are as reconstructed and unprocessed instrument and consists of the image data including the radiometric coefficient and the geometric coefficient and other auxiliary data. While, Level-1B data are generated by applying these coefficients for radiometric calibration and geometric sampling. In this study, Level-1 A, V003 data series were considered for different time periods depending on the selected image scene; and therefore, 10 images were retrieved (Table 1). Thermal Infrared bands were also adopted to detect thermal differentiation for the linear surficial features that evidencing buried features (i.e., paleodrainages).

Consequently, ASTER satellite images were mosaicked using ERDAS Imagine-14 software. The five thermal infrared (TIR) bands were then corrected for atmospheric effect using an algorithm that is similar to the in-scene atmospheric compensation algorithm or *ISAC (in-scene atmospheric correction).* The algorithm initiates by identifying the spectral band that returns maximum brightness temperature for most pixels, which is set as the reference band. There are several digital procedures were applied on ASTER images for a better discrimination of features including: band combination, edge detection, filtering, stretching histograms equalizing, etc. For instant, the stretching histograms equalizing shows a graphical representation of the intensity distribution that represented by the number of pixels for each intensity value. It enables improving the contracts of the images, by spreading out the most frequent intensity values, i.e. stretching out the intensity range of the image.

Multi-looking process improves the radiometric resolution of the Single Look Complex (SLC) image where multiple looks Intensity images are generated by averaging over range and/or azimuth resolution cells. The product represents an intensity image, composed of squared pixels where the ground range resolution and the pixel spacing in azimuth are considered. Retrieved images from the PALSAR sensor are often characterized by speckle (noise) due to statistical fluctuation associated with the radar reflectivity of separate pixel per scene. PALSAR data is received by Level 1.0 Raw which is extracted to produce a SLC file. SAR data over the study area was acquired by the ALOS-PALSAR-2 sensor at L-band (λ 25 cm) with pixel spacing of 6.25 m in fine-beam dual-polarization mode (horizontal-horizontal- HH & horizontal-vertical-HV), in an ascending orbit with off-nadir angle of 28.6°.The data was pre-processed with several steps including: removal of antenna variation effects, speckle filtering by means of adaptive Lee-Sigma, Frost and Gamma-MAP filters, Ortho-rectification and Re-projection, Radiometric calibration through converting (DN) values into Backscatter Intensity in decibel format (dB). The processing of ALOS PALSAR images followed a number of digital processes, namely: (1) Radiometric correction, (2) Terrain correction and (3) HH & HV polarization. The geocoded (i.e., georeferenced) images are saved as Geo-Tiffs. Products include 8 and 32 bit Geo-Tiffs Which are re-projected from their default to the appropriate spatial reference system in ERDAS Imagine.

SRTM DEM was primarily processed to extract various watercourses with diverse dimensional aspects (i.e., stream networks). The advantage of using more than one type of remotely sensing product with different optical and spectral signatures was obvious in this study. Even for the same type of satellite images (e.g., ASTER), various digital procedures were applied to confirm the presence of the linear features which are totally absent in the streams networks extracted from SRTM DEM and from topographic maps.

Given that all streams have been extracted from the SRTM DEM; therefore, the existing drainage systems (i.e., stream networks) can be drawn for the study area. Consequently, ASTER images enable us to identifying the wet linear surfaces by using the thermal bands, and these surfaces evidence the presence of covered wet horizons which are probably due to the paleodrainages. Further on, ALOS PALSAR images will be utilized due to their capability of terrain surface penetration and this enabled detecting buried features Based on the processing of the three mentioned satellite images, comparative analysis was carried between the existing stream networks, the wet linear surfaces along and the anomalous linear features which were extracted from SRTM DEM, ASTER and ALOS PALSAR images; respectively (Fig. 2). The resultant evidences the existence of potential paleodrainages, and this was reached after using the previous mentioned digital procedures on ERDAS Imagine and Arc-GIS software.

### Field verification

**Fig. 3:**
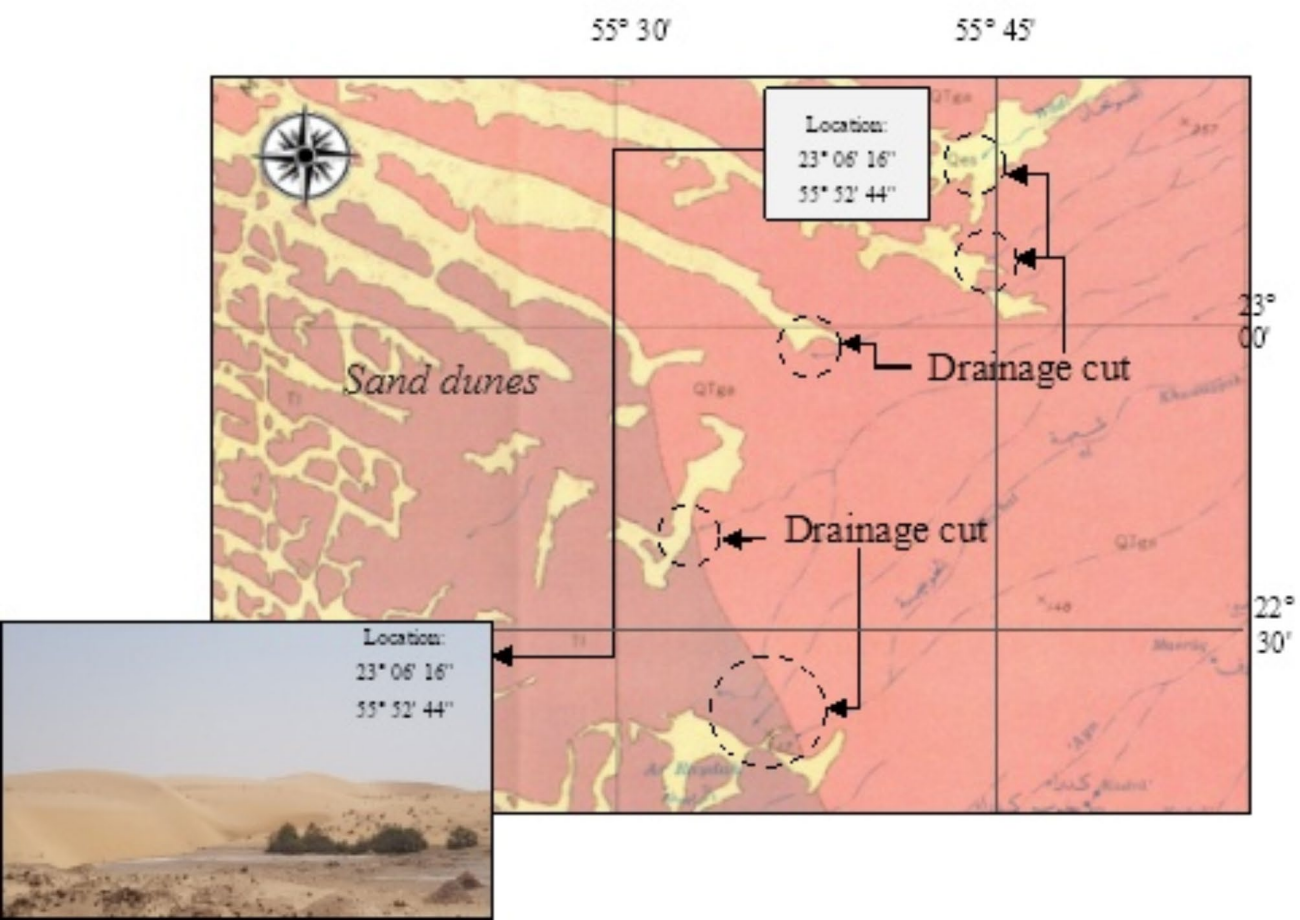
Abrupt cut of drainages when reaching loose materials (e.g., sands) as revealed on a geologic map (Elberg et al., 1963).

Abnormal drainage patterns are often observed on topographic maps and also on the extracted from high-resolution (30 m) DEMs. These comprise abrupt drainage cut notably when loose materials (e.g., sand and alluvial deposits) exist (Fig. 3), but they are detected on the processed satellite images. Field truthening was carried out to assure the existence or to find out any key feature evidencing ancient hydro-geomorphological processes in such cases. Usually, field observations and ground-truthening of paleodrainages show changes in the wetness of surface land materials, while vegetation are considered a potent field approach^44^. The emphasis of this study was on the results obtained from processed satellite data rather than applying in-depth field surveys such as geophysical surveys. This was also the case in several previous studies applied on ancient (& hidden) hydro-geomorphological features^15,26,45,46^. For this work, the following field verification methods were applied:

1. *Surficial clues*: These are well pronounced in the delineated drainage systems which sometimes show abrupt disappearance, but this is not always the case and some drainages merely covered by moveable materials such as loos sand (example in Fig. 3), and this is well pronounced in Saudi Arabia^10^. Field observations have been carried out to inspect any clues evidencing the presence of hidden hydro-geomorphological features. In the study area, the most identified surficial clues include: (i) Elongated water bodies (& wet soil horizons) that abruptly exist in desert regions, (ii) Sudden longitudinal strips of vegetation cover in the dry lands, (iii) Incised streams that abruptly terminated (iv) Saline soil and linear salt crusts at a range from the coast (e.g., several tens of kilometers).
2. *Trenchin*g: This was applied manually, whenever it was possible, to verify the litholofacies characteristics of the beneath materials. The depth of the dug trenches in this study ranged from 0.5 m to 1.80 m, with various widths that allows picking located materials. Moreover, we applied our investigation along rock outcrops whenever that was possible. Target materials were mainly the ancient alluvial deposits (e.g., cobbles, pebbles, organic matter, etc.) and heterolithic sandstone, silty and shale sequences.
3. *Auger sampling*: This was also applied in the field to pick up samples notably those where wet horizons occur. The auger, with coring diameter of 2.4” (~ 6 cm), was capable to sample sediments between of 40–80 cm depth. The target materials are the same as in the trenching method, where the shallow paleo-deposits and sedimentological sequences were investigated.
4. *Well logs*: This tackles available data from groundwater boreholes which were dug into shallow aquifers (i.e., few meters’ depth), and their location matches with the extension of an identified paleodrainages. The investigated logs often showed alluvial deposits with a wide variety of sediments grain size (e.g., fine clay - cobbles), while groundwater depth was almost shallow (i.e., 15–40 m).

## Results

### Remote sensing-based evidences

**Fig. 4:**
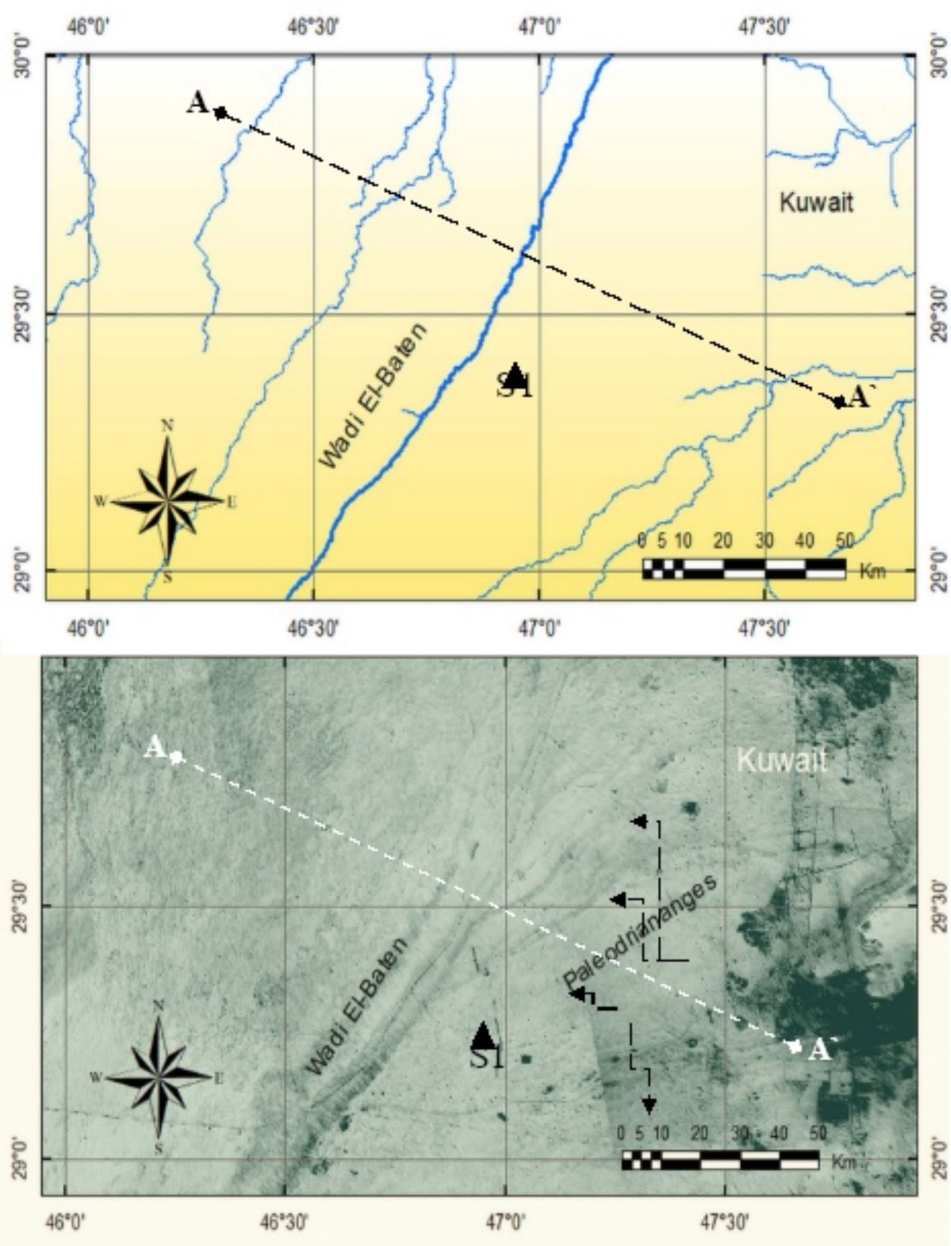
Two images for the same area. The image above shows the drainages as existed on SRTM DEM; while the image below is a processed ALOS PALSAR image showing traces of delta-like shape paleodrainages (D*Pl*); but they do not coincide with the current drainages in the SRTM DEM (image above). Triangle at point S1 is the site where the stratigraphic sequence was investigated.

**Fig. 5:**
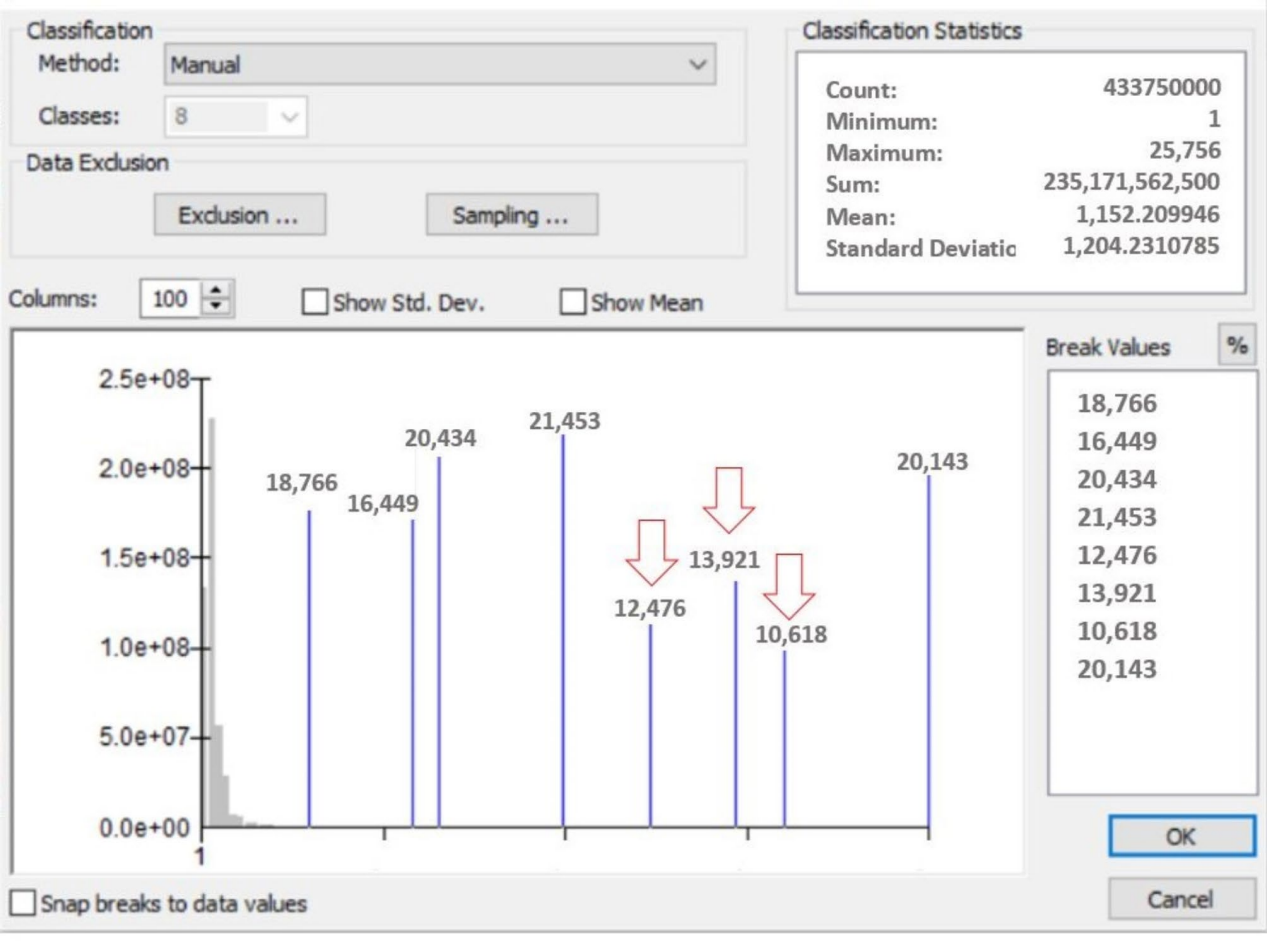
Example of applied spectral analysis on ALOS PALSAR images for the transverse AA` in Fig. 4. The lower spectral values (indicated by red arrows) show the presence of invisible drainages (D*Pl*).

Linear geomorphological features with anomalous drainage patterns have been observed on various satellite images. They were dually analyzed in this study by applying digital advantages on more than one type of satellite images. The detected features were not considered as paleodrainages until they were clearly distinguishable by the viewer. Digital advantages in the ERDAS Imagine-14 software were applied for these images. This was also supported, in some cases, by field verification considering the methods and features mentioned in Sect. 2.3. The performed digital applications enabled calculating the horizontal dimensions of the recognized paleodrainages; and then, we presumed the delineation of the incised stream and visualize their entire orientation and behavior. Therefore, the following types of paleodrainages were detected:

1. Delta-like shape paleodrainages (D*Pl*):

These are buried watercourses with multiple tributaries representing delta-like shape drainages that might belong to ancient primary rivers (i.e., paleo-rivers) where they extend several hundreds of kilometers over playa landforms (Fig. 5). The analyzed D*Pl* in this study neither appear as existing streams on the topographic and geologic maps nor on the extracted drainages from SRTM DEM (Fig. 4), but they are obviously observed on the processed ALOS PALSAR images. The most typical example is that exists along Wadi El-Baten to the NE of Saudi Arabia where plume–like shape drainages appears on the processed ALOS PALSAR images, but they cannot be distinguished on topographic and geologic maps (Fig. 4). These paleodrainages were probable large delta with an average width range between 140 and 180 km. The general trend is mainly in the SW-NE direction evidencing low slope from the Arabian Shield^10^.

The cover-up materials are mostly of sand dunes that burdening D*Pl*, and they are usually with considerable thickness. These cover-up materials, are composed mainly of Quaternary deposits with unconsolidated deposits and variety of sediments, and sometimes they mixed silt and sand, which are overlying Tertiary sandstone sediments and rocks that in many instances intervene sandy marl and sand limestone. The thickness of the cover-up materials is often diverse, and it ranges between tens of centimeters to more than 5 m and the latter is found where sand dunes are formed. The region where the cover-up materials exist is dominant by the aeolian erosion, sand drifts and mass movements.

The observed D*Pl* on the processed ALOS PALSAR images has been also verified using digital advantages on ERDAS Imagie-14 software, where classification method was performed based on the spectral analysis of the image features; and therefore, the multispectral data was used for categorization of terrestrial objects. Figure 5 shows an example of an image classification across the traverse selected on Fig. 4 which cross along different drainages existed on the image, including the buried paleodrainages. It is clear form Fig. 5 that the paleodrainages (D*Pl*) are existed with different spectral values., and this evidences the presence of these ancient watercourses in the region. The spectral signatures of these paleodrainages have almost close values, but they are totally different from the values of existing drainages (Fig. 5).

**Fig. 6:**
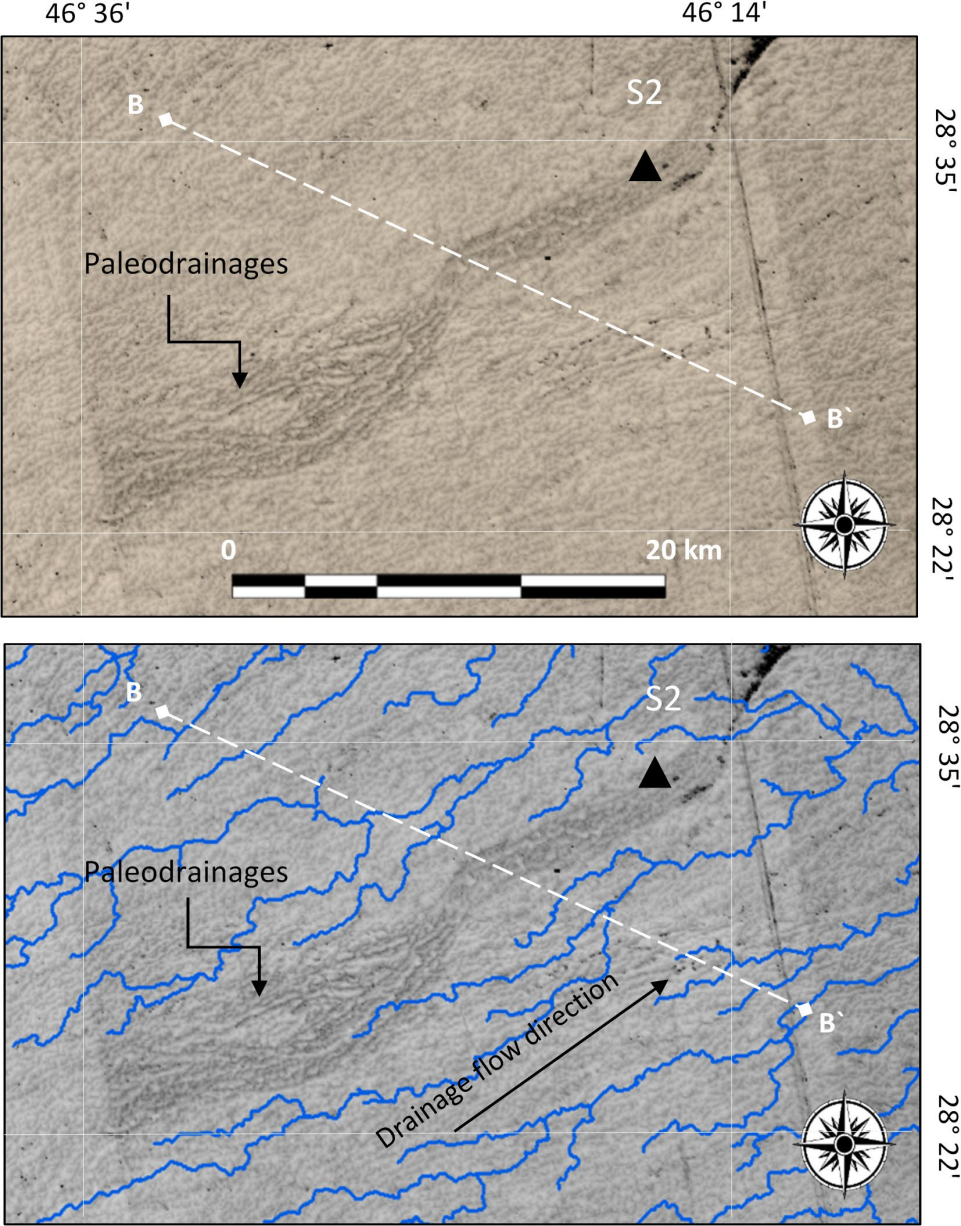
The image above shows a shaded figure for an odd paleodrainage (O*Pl*) as shown on the processed ASTER image, but not in the existing drainage system. The same ASTER image was used below after projecting the current drainages extracted from STRM DEM. There is no coincidence between the O*Pl* and the current drainages evidencing an invisible paleodrainage (O*Pl*). Triangle at point S2 is the site where the stratigraphic sequence was investigated.

2. Odd paleodrainages (OPl):

These represent isolated drainages which appear, only on the processed thermal satellite images (Fig. 6), as traces of linear features (i.e., odd buried watercourses). They are characterized by considerable areal extent (e.g., tens of kilometers) and width. The existence of these buried type of drainages, with relatively large scale and such orientation can be attributed to high runoff (i.e., large flow) of water in the past which is probably resulted from the paleo-climatic changes. This type of paleodrainage has the same flow trend of the existing drainages which is probably due to low slopes that might be attributed to the aeolian processes (Fig. 6). The case of cutting cross between old and new drainage systems has been well demonstrated by El-Bastawesy (2014)^10^.

The cover-up materials in areas with O*Pl* is attributed to the Quaternary age and they are composed mainly of silt and fine sediments including caliche-like and gypsiferous deposits. The thickness of the cover-up materials ranges between few centimeters up to more than 4 m where paleodrainages covered by this thickness is a bit difficult to be detected by satellite images even the microwave sensors.

**Fig. 7:**
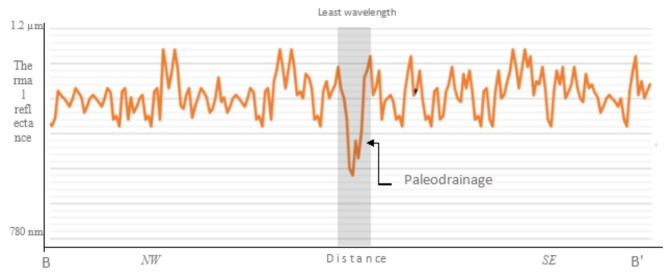
Example for the performance of thermal differentiation on Aster image processed with ERDAS Imagine along the traverse BB’ shown in Fig. 6. Least thermal reflectance occurs along the identified O*Pl* evidencing more wetness due to the presence of porous and permeable zones, probably the O*Pl.*

The detected O*Pl* on the processed ASTER satellite images were also evidenced using thermal band on ASTER (90 m) to detect any thermal differentiation in ERDAS Imagne-14 software. The resulted thermal reflectance has been performed along the BB’ traverse (plotted in Fig. 6). Therefore, obvious decline in the wavelength has been determined (Fig. 7), showing decreasing in the wavelength on the traverse crosses along the observed on the ASTER image of Fig. 6, and this gives an evidence for the changing in wetness due to porous and permeable zone due to the presence of O*Pl*.

3. Paleo-lakes (plL)

**Fig. 8:**
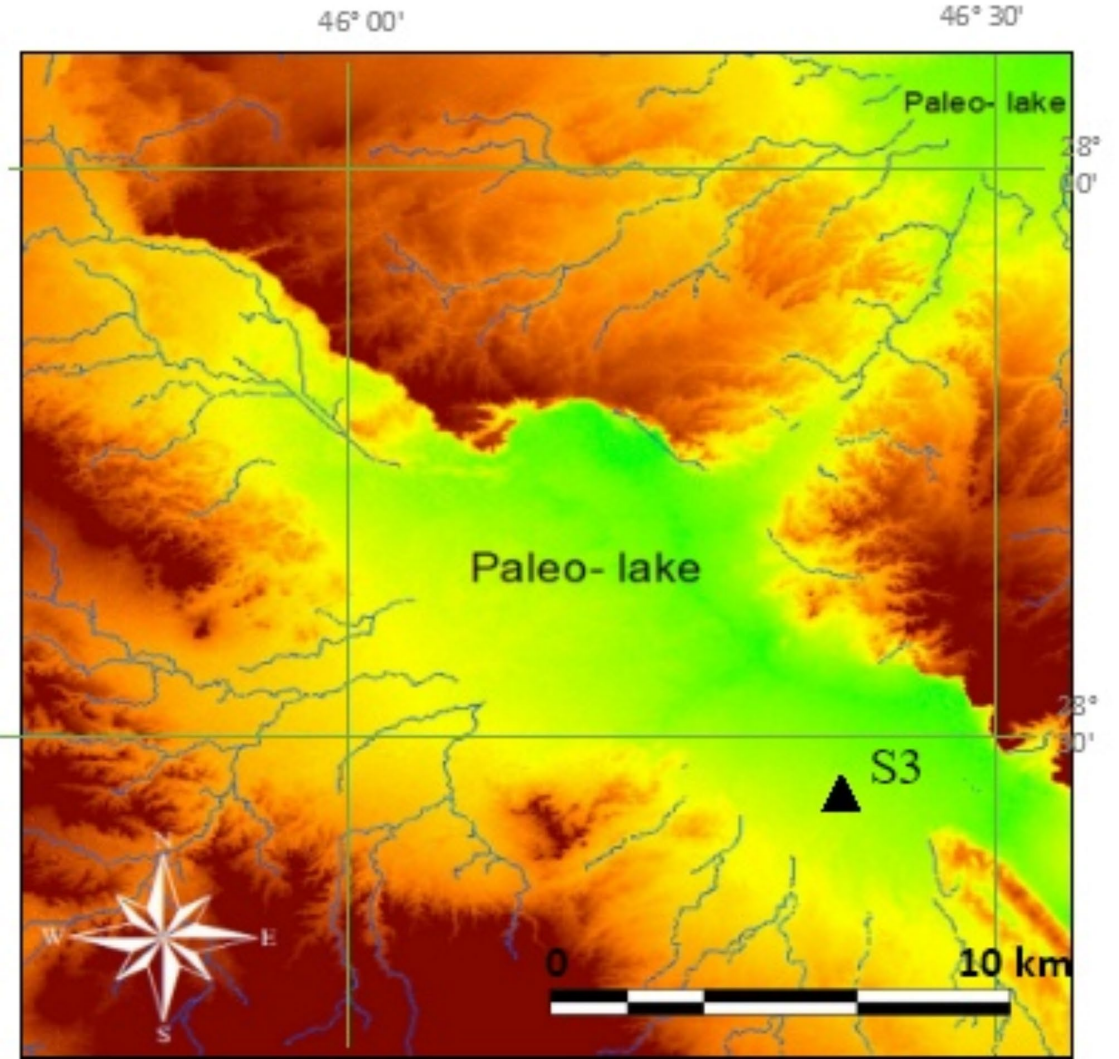
SRTM DRM showing intermittent drainages with a unified orientation and alignment towards a depression-like shape indicating an ancient paleo-lakes (*Pl*L) occurred in the regions. Triangle at point S3 is the site where the stratigraphic sequence was investigated.

There are numerous observations noted on the SRTM DEM and even in the topographic and geologic maps where the subsequent drainages are abruptly quitting (i.e., disappear), and this is well pronounced either in low-lands and depression-like terrain or sometime into loos sand dunes; especially where eroded detrital sediments (i.e., aeolian, alluvial and colluvial deposits) exist (an example was shown in Fig. 3) with thickness ranging between 0.5 m and 5 m. The delineation of the existing drainages on SRTM DEM reveals drainage systems that orientated and aligned towards a unified direction and then they join at a define zone usually a depression, but a sudden extinction of these tributaries is occurred when they reach such a depression or sand dunes (Fig. 8).

The configuration of these paleodrainages at the upstream region is dominant with consequent and obsequent streams^10^, which invisibly rejoined (i.e., tens of square kilometers) evidences that an ancient lake was occurred there in the past, but the vanishing of water flow made a region with intermittent drainages which obviously showing the input and output into/from the probable paleo-lake (*Pl*L), which had been dried then. There is thick accumulation (i.e., up to 5 m) of loos sand and sand dunes where *Pl*L occurs, and the thickness becomes moderate and thin at the edges of the depressions of *Pl*L. The cover-up materials belong to Tertiary rocks where they are composed of gravel, limestone pebbles and cobbles which are locally cemented by ferruginous caliche.

4. Paleodrainages induced by flow diversion (*Pl*FD)

**Fig. 9:**
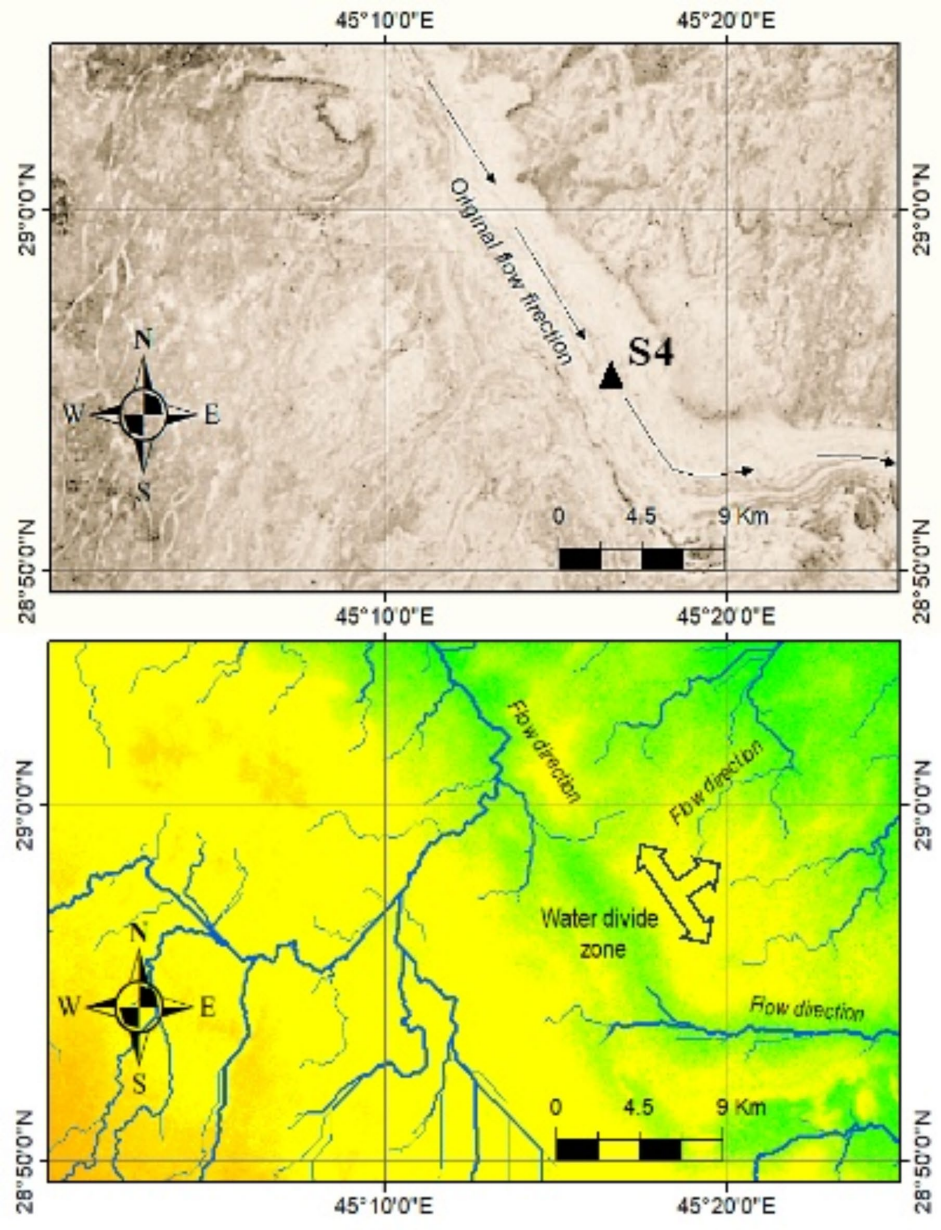
Example of ALOS PALSAR image (above) showing paleodrainages induced by flow diversion (*Pl*FD), and the existing (current) drainage network extracted from SRTM DEM (below) where three main flow directions and obvious water divide zone. Triangle at point S4 is the site where the stratigraphic sequence was investigated.

There are configured dry drainages recognized and characterized by tens of kilometers length and valley cross-section exceeding 2 km in many regions of Saudi Arabia^5^. These are connected at the upstream part with consequent and obsequent streams, but consequently dried. In this respect, the use of SRTM DEM supported by ALOS PALSAR images interpreted the probable delineation of paleodrainages (*Pl*FD) as a continuation for the existing ones. They can be also interpreted by the geometric and morphometric analysis; especially that a water divide zones can be obviously identified and it is potential for *Pl*FD (Fig. 9).

The geological framework of *Pl*FD is mainly attributed to orogenic tectonism resulted in a geologic structure such as graben, uplift, faulting, etc. that later on altered the hydrologic system and then diverted the runoff water into different directions than the consequent stream direction. This geomorphologic phenomenon has been noted in several region of the Arabian Peninsula where this type of paleodrainages was described as “anomalous streams”^22^.

The cover-up materials where *Pl*FD occurs is dominant with eroded sediments and mostly of coarse sediments mixed with large rocks bodies, conglomerates and rock debris (colluviums) that stuffed the paleodrainages. In the study area, mixed quartz gravel sheets with some limestone pebbles of the Quaternary age are embedded with clastic limestone and marly od the Tertiary rocks. Hence, moderate to thick cover-up materials often occur and ranging from zero to several meters thick, and this is normal because no flow occurs along the *Pl*FD.

### Field characterization

Field study has been carried out to characterize the identified paleodrainages in four sites (S1-S4) which have selected to determine the type of materials stuffed into the identified paleodrainages on satellite images. The field verification followed one method (or more) as mentioned in Sect. 2.3; therefore, the following were resulted:

1. Delta-like shape paleodrainages (D*Pl*)

**Fig. 10:**
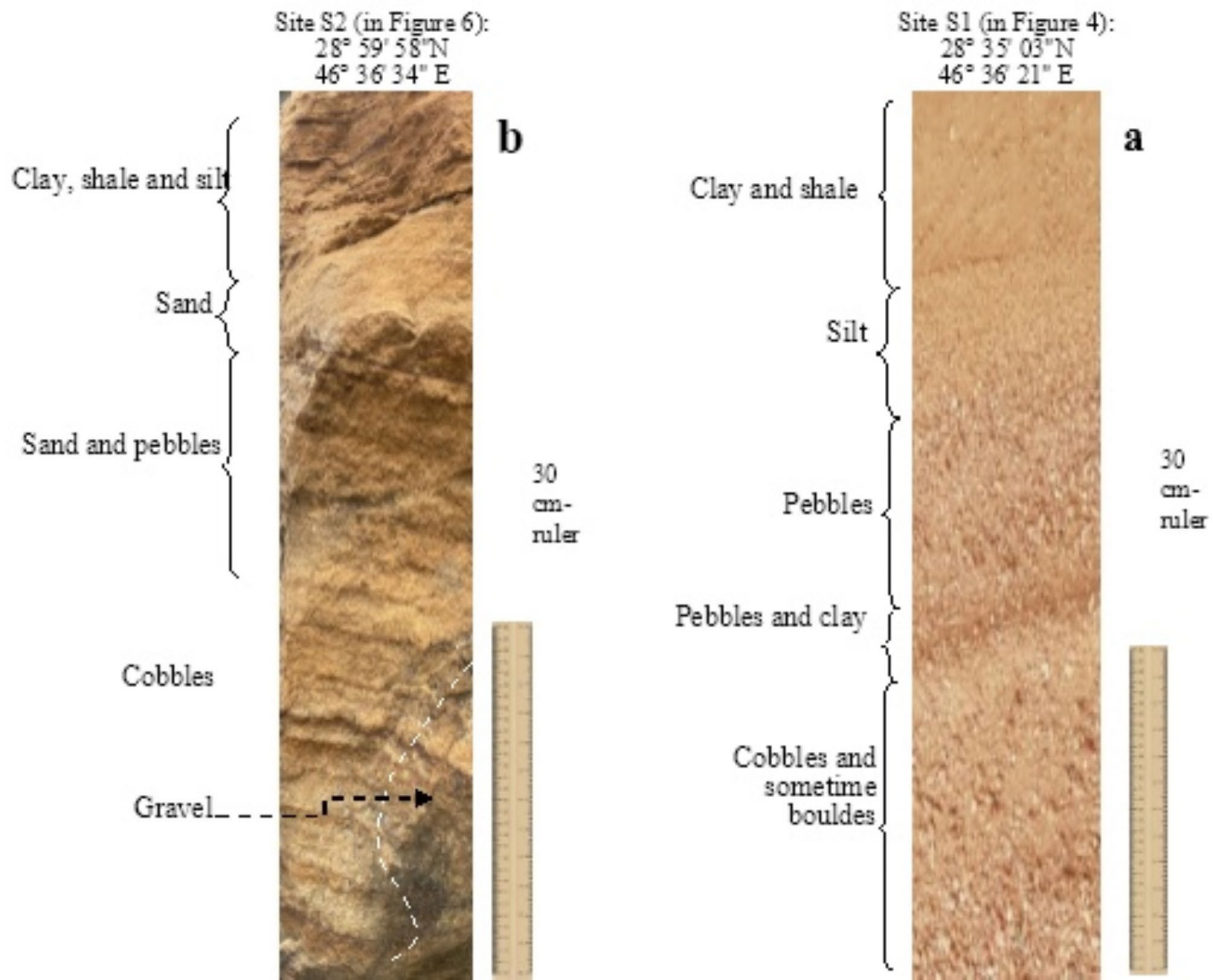
Representative stratigraphic sections on DPl and OPl as identified in the filed investigations for sites S1 and S2.

In-situ investigation on the stratigraphic sequence for the identified D*Pl* shows noticeable depositional environments with dominant coarse and rounded sediments (i.e., pebbles, cobbles and sometimes boulders) as shown in Fig. 10a. This indicates that large loads of runoff water along these drainages occurred in the past, and they were probably on a river scale. The studied stratigraphic sequence obviously shows transitional decrease in the sediment’s grain size upward where fine grains sediments (e.g., clay, silt, etc.) exist into the upper parts of the succession. This is probably due to the changing climatic conditions with decreased rainfall rate eventually resulted in the dryness of these drainages.

D*Pl* were typically found to the northern part of Wadi El-Baten which spans from Wadi El-Rumah in the middle of Saudi Arabia to the N-NE direction, and they geomorphologically formed the Kuwait and part of Iraq territories. Even though, they were not given a specific nomenclature, there are several studies mentioned similar paleodrainages^10,19,47–50^.

2. Odd paleodrainages (O*Pl)*

Field verification for the depositional environment of the O*Pl* shows dominant fine sediments (e.g., clay, shale and silt) which are interbedded, at various depth, with coarser sediments of pebbles and cobbles (Fig. 10b). This in turn evidences that the runoff was along wide floodplain of a large-scale watercourse (i.e., river) occurred in relatively flat area with saturated and fertile soil. A good example is from Eastern Libya, where several aspects of large-scale odd paleodrainages (O*Pl*) were detected, and the most significant one is called Al-Kufrah River (i.e., buried watercourse) with about 900 km length and 400.000 km^2^ paleo-watershed^24,26^.

3. Paleo-lakes (*Pl*L)

**Fig. 11:**
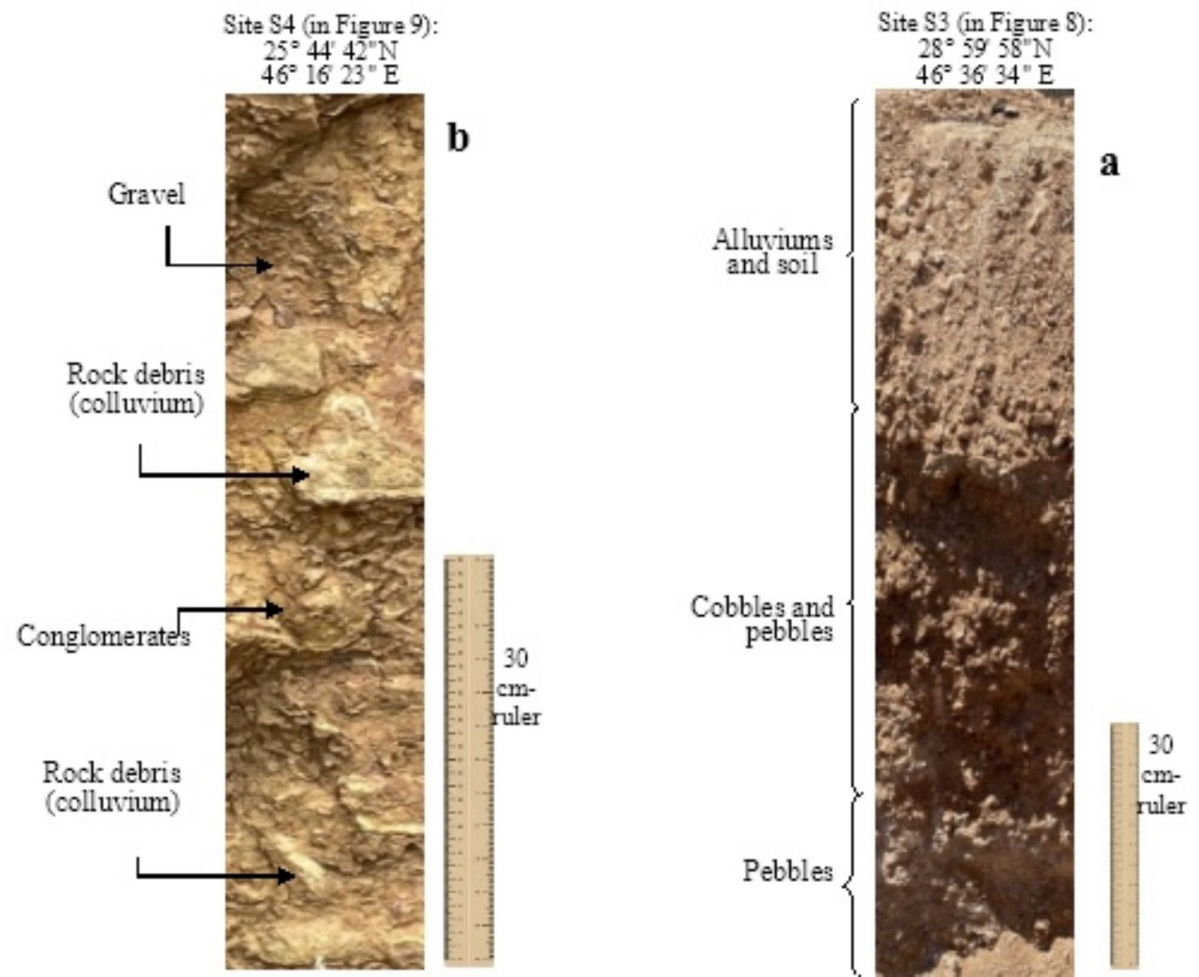
Representative stratigraphic sections on PlL and PlFD as identified in the filed investigations for sites S3 and S4.

Numerous terrain surfaces with depression-like shape and relatively large dimensions (i.e., few square kilometers length) were deduced on the processed satellite images (e.g., SRTM DEM) on the studied area, and these buried *Pl*L form irregular geographic patches where drainages disappear there. Field study shows that these sites are found with dominant argillaceous materials and mainly fine to very fine sediments including clay and silt with many inclusions of organic matter that distribute vertically and horizontally along the dug cross section, while pebbles also exist (Fig. 11a). However, alluvial deposits are found on the top of the section as a result of continuous erosion where sand is dominant due to the existing aeolian erosion in the region. It is worth mentioning that the argillaceous materials found with tangible wetness, and the facies of the stuffed deposits are totally different from the surrounding cover-up materials indicating upnormal geomorphologic feature.

4. Paleodrainages induced by flow diversion (*Pl*FD)

The sites where *Pl*FD were identified showed dominant eroded deposits, and mostly of coarse sediments mixed with large rock specimens, conglomerates and rock debris (colluviums) that stuffed into these paleodrainages. The investigation of stratigraphic sequences reveals a clear heterogeneity in the existed materials including rock debris and colluvial deposits that irregularly embedded (Fig. 11b). Colluvial rock specimens are sometimes connected with gravel and with another finer deposits forming the matrix material. The angular edges of the rock samples evidence the presence of tectonic processes. Sediments and rock debris in the proposed *Pl*FD evidence that they are different from the cove-up materials located in the investigated site.

Similar paleodrainages were mentioned in some studies where they either focused on the materials forming these g features^51^, the geomorphologic and hydrological processes and evolution^52,53^, or the sedimentological characteristics of these drainages^54,55^.

The identified paleodrainages in the study area are mainly stuffed with alluvial and fluvial deposits with a variety of runoff sediments and other surficial processes which indicate that these buried watercourses were filled with water in the past. This in turn evidences that the identified buried drainages are “paleodrainages” in type. In this respect, four main types of these paleodrainages were identified and characterized as shown in Table 2.

## Discussion

Studies on paleodrainages are often applied in-situ using filed surveys (e.g., geophysical investigation), and recently many studies adopted satellite images as a significant tool to detect these geomorphological and hydrogeological features with wider areal coverage^20,38^. However, performed studies using satellite images tackled various issues, such as the origin and evolution of paleodrainages^50,56^; their evidences; paleodrainages and hydrology^10,36,38^; climate-related paleodrainages^53,57,58^; paleodrainages and ore deposits^59^, groundwater mechanism related to paleodrainages^60–62^. Nevertheless, all these studies did not show any classification, while, this has been considered in this study which utilized a miscellany of satellite images with diverse optical and spectral characteristics.

This study utilized various satellite images, and each image was processed using various digital advantages. Thus, each type of images was allocated for specific scope, and then STRM DEM was generated to draw the current (existing) stream networks, while the linear features detected by ASTER and ALOS PALSAR images. In this respect, the combination of various satellite observations was not performed in previous studies^38,63,64^, but all processed one type of satellite images and the resulted drainages were not investigated in the field to ensure if the they belong to ancient watercourses (i.e., paleodrainages). However, inaccurate interpretation may occur due to the diverse surficial post-processes that might affect these features, such as weathering, extreme climatic conditions as well as the interference of human activities. In this respects, the potential paleodrainages with diverse features (e.g., dimensions, orientation, patterns, etc.) evidenced different climatic conditions and geological processes occurred in the past.

**Table 2:** Major characteristics of the identified paleodrainages in the studied area in Saudi Arabia.

Identified paleodrainages	Potential controlling factor	Depositional environment	Cover-up materials
Delta-like shape paleodrainages (D*Pl*)	Changing climatic conditions with slight decrease in rainfall.	Fluvial environment of deposition with dominant sedimentation of coarse-grained sediments that traditionally lowering upward and indicating consequent decrease in flow rate in the top.	- Unconsolidated Quaternary deposits of silt and sand that overlying the Tertiary clastic limestone and marl of the rocks composed of sandstone, sandy marl and sand limestone. - Thickness ranges from few centimeters to more than 5 m.
Odd paleodrainages (O*Pl*)	Almost due to the changing climatic conditions that may result these paleodrainages	The dominant fine sediments evidence calm energy water flow interrupted by increased water level sometime due to sudden rainfall intensity.	- Quaternary deposits of silt and caliche-like sediments with gypsiferous deposits. - The thickness ranges between 0.5 m up to more than 3–4 m.
Paleo-lakes (*Pl*L)	Geomorphological processes (aeolian) associated with geological potential deformations.	The heterogeneity of fine (clay) and rounded sediments (pebbles and cobbles) with some angular edged grains of gravel evidences geological events occurred during regular water flow.	- Thick sand dunes (few meters) occur where *Pl*L in sand dues are located. For those *Pl*L in low-land, there are moderate to thin accumulated sediments of Tertiary rocks composed of gravel and limestone pebbles and cobbles locally cemented by ferruginous caliche. - The thickness of the cover-up materials in low-lands are mainly between 0.5 to 5 m.
Paleodrainages induced by flow diversion (*Pl*FD)	Large-scale tectonic activity with secondary rock deformation.	*Pl*FD in low-lands and depressions are stuffed with eroded coarse sediments, conglomerates and rock debris mainly of colluvial deposits indicating dominant mass movements and erosion along slopes.	Mixed quartz gravel sheets with some limestone pebbles of the Quaternary sediments embedded with clastic limestone and marly od the Tertiary rocks. - Thin cover-up materials may exceed several meters in many instances.

The geology and geomorphology of the study area, a selected geomorphologic region of the Arabian Peninsula, was investigated and it was obvious that this area was subjected to several natural changes including climatic and geologic ones that affected the existed geomorphological and hydrogeological systems. This is well pronounced from the buried drainages into desert areas. The hydrogeological and geomorphologic characteristics of the identified paleodrainages were interpreted depending mainly on observations from satellite images and from the field verification.

In this study, it was well understood that not all paleodrainages can be detected by the same satellite images due to the spectral properties of these satellites, as well as the materials stuffed in these drainages and their hydrologic and spectral characteristics. In addition, field observations evidenced that paleodrainages are controlled by several surficial processes occurred over time, their depth from the terrain surface, thickness and type of the cover-up materials as well as wetness property.

The depositional environments of the investigated stratigraphic sequences clearly show that these sequences belong to extinct watercourses that were filled with water in the past. In addition, the diversity in the sediment’s properties and mainly the grain size, shape and stratification evidenced different flow energy/regime as a result of climatic controls; while the presence of gravel with angular surfaces (i.e., colluvial deposits) in some localities pointed out to the geologic events represented mainly by fracture systems (e.g., faulting) that resulted in mass movement.

Based on the detailed literature review while this study, it was well understood that the detection of paleodrainages remains with no common scientific background, and challenges often exist; especially that these ancient watercourses have a large (& longitudinal) geographic extent (as for all identified paleodrainages in this study), and they are sometimes found buried with thick accumulation of sediments (such as in the D*Pl*,* Pl*L and *Pl*/FD), while others are observed with anomalous behavior of the recent drainages (such as *Pl*L and *Pl*/FD). These challenges in recognizing paleodrainages are usually addressed by combining the use of space observation from more than one type of satellites, investigating the overall behavior of recent drainage systems, as well as the field inspection.

## Conclusion

Satellite images have been developed lately as a tool in many applications for discovering Earth’s surface and the surficial processes; one of these applications is the exploration of hidden Earth’s features, and more specifically the dry buried watercourses (i.e., paleodrainages) that have been studies by many researchers. This study utilized the STRM DEM, which is an elevation dataset collected by satellite and charcaterized by stereoscopic visualization and capable to identify terrain topography joined with the extraction of drainage systems; while PALSAR is a sensor mounted on ALOS satellite and it enables penetrating the subsurface rock layers to detect buried features. ASTER images can perform thermal differentiation to detect wet horizons.

This study focuses on detecting these features in a large selected area from Saudi Arabia (143.000 km^2^). The novelty in this study includes the integration of multi-remote sensing products followed by field verification. During our literature review studies on paleodrainages, very few studies investigated the lithological facies of the stuffed materials in paleodrainages, and this added another novelty for this study.

Moreover, this study classified four types of the paleodrainages, and this classification was not tackled in previous studies, but emphasis was only on describing one type of the recognized paleodrainages. The four identified types of paleodrainages have been characterized from the geomorphological, hydrological and depositional point of view.

Buried terrain features were often tackled as a part of historical geology and geochronological assessment, but lately these features have been described from the economical point of view; and therefore, they have been primarily mentioned as a source of ore deposits such as placer gold and other metallic ores^65^. However, recent concerns are on water resources development; especially with the exacerbated climate change and the resulted water scarcity; and thus, paleodrainages are sought as a source for water^28,66^. This is significant for a region like Saudi Arabia where precipitation does not exceed 150 and the major groundwater aquifers store only fossil water; besides seawater intrusion is widespread in many coastal zones of the entire Arabian Peninsula.

In a recent study still under implementation by the author^30^, paleodrainages are considered as promising hydrological features, because they are potential for groundwater storage (i.e., shallow aquifers in alluvial deposits), as well as they can be potential sites for groundwater artificial recharges (GWAR); especially that positioning suitable sites to GWAR remains a challenge in many regions^67–69^. Thus, paleodrainages can be suitable localities where the flood water in wadis such as in Saudi Arabia, can be recharged to feed the beneath aquiferous rock formations. Another point of concern was raised in this study, it is the extent of saline soil and Sabkhas at a range into land, and this is well pronounced in several localities of Saudi Arabia including the study area, and certainly in Ad-Dammam Region where large number of Sabkhas exist even tens kilometers from the coast and resulting in unfavorable and bad lands.

## Data Availability

The datasets used and/or analysed during the current study available from the corresponding author on reasonable request.

## Declarations

The datasets used and/or analysed during the current study available from the corresponding author on reasonable request.

## References

[1] GAMEP (The General Authority of Meteorology and Environmental Protection). Information and statistics. (2021). https://www.mewa.gov.sa/en/InformationCenter/Pages/default.aspx

[2] Baban, S. The Suitability of Satellite remote sensing and gis technologies for mapping, monitoring and managing water resources in the middle east. in *Satellite Monitoring of water resources in the Middle East* (eds Shaban, A.), vol. 1, 29–47 (Springer Nature, 2022).

[3] Wehbe, Y. Unraveling the spatio-temporal dynamics of satellite-inferred water resources in the Arabian Peninsula. in *Satellite Monitoring of Water Resources in the Middle East* (eds Shaban) (Springer, 2022).

[4] IPCC., The Working Group II contribution to the IPCC 6th Assessment Report on climate change 2022: Impacts, adaptation and vulnerability. (2022).

[5] Al Saud, M. *Flood Control Management for the City and Surroundings of Jeddah, Saudi Arabia*. 177 (Springer, 2015). ISBN13: 978-94-017-9660-6.

[6] Shaban, A. *Water Resources of Lebanon*. 229 (Springer Sci. Publisher, 2020). ISBN 978-3-030-48716-4.

[7] Hötzl, H. et al. Wadi Ad Dawasir and its Hinterland. in *Quaternary Period in Saudi Arabia* (eds Al-Sayari, S. S. & Zötl, J. G.). (Springer, Vienna, 1978). 10.1007/978-3-7091-8494-3_10.

[8] Jawad, S., Al-Sulaimi, A. & Pitty, F. Origin and depositional model of Wadi Al-Batin and its associated alluvial fan, Saudi Arabia and Kuwait. *Sed. Geol.* **97** (3–4), 203–229. 10.1016/0037-0738(95)00011-V (1999).

[9] EOS. Arid Arabian Peninsula Is Tapping into Vast Groundwater Reserves. https://eos.org/articles/arid-arabian-peninsula-is-tapping-into-vast-groundwater-reserves (2019).

[10] El-Bastawesy, M. The geomorphological and hydrogeological evidences for a Holocene deluge in Arabia. *Arab. J. Geosci.* **8**, 2577–2586. 10.1007/s12517-014-1396-9 (2014).

[11] Woor, S., Buckland, C., Parton, A. & Thomas, D. Assessing the robustness of geochronological records from the Arabian Peninsula: A new synthesis of the last 20 ka. *Glob Planet. Chan*. **209**, 103748. 10.1016/j.gloplacha.2022.103748 (2022).

[12] Robinson, C. et al. Use of radar data to delineate palaeodrainage flow directions in the Selima sand sheet, Eastern Sahara. *J. Photogrammetric Eng. Remote Sens.* **66**, 745–753 (2000).

[13] Robinson, C., El-Baz, F., Al-Saud, M. & Jeon, B. Use of radar data to delineate paleodrainages leading to the Kufra oasis in the eastern Sahara. *J. Afr. Earth Sci.* **44**, 229–240 (2006).

[14] Almulla, S., Albadran, B. & Al-Ali, A. Application of remote sensing techniques to Map the paleochannels of Shatt Al-Arab and Khor Al-Zubair, Southern Iraq. *Marsh Bull.* **6** (1), 23–31 (2011).

[15] Ghoneim, E., Benedetti, M. & El-Baz, F. An integrated remote sensing and GIS analysis of the Kufrah Paleoriver, Eastern Sahara. *Geomorph* **139–140**, 242–257 (2012).

[16] Zaidi, F., Nazzal, Y., Ahmed, I., Naeem, N. & Jafri, J. Identification of potential artificial groundwater recharge zones in Northwestern Saudi Arabia using GIS and boolean logic. *J. Afri Ear Sci.* **111** (Nov. 2015), 256–169 (2015).

[17] Rosenberg, T. M. et al. Middle and late pleistocene humid periods recorded in palaeolake deposits of the Nafud desert, Saudi Arabia. *J. Quart. Scien Revi*. **70**, 109–123 (2013).

[18] Emil, M. et al. *Reconstruction of the Paleohydrological Setting of the Rub Al Khali, Saudi Arabia* (AGU, Fall Meeting, 2013). Abstract id. H43G-1543.

[19] Breeze, P. S. et al. Remote sensing and GIS techniques for reconstructing arabian palaeohydrology and identifying archaeological sites. *J. Quart. Inter.* **382**, 98–119 (2015).

[20] Ul Islam, Z., Iqbal, J., Kha, J. & Qazi, W. Paleochannel delineation using landsat 8 OLI and Envisat ASAR image fusion techniques in Cholistan desert, Pakistan. *J. Appl. Rem. Sen * (4), 046001. 10.1117/1.JRS.10.046001 (2016).

[21] Howard, A. D. Drainage analysis in geologic interpretations: a summation. *Am. Association Petroleum Geol. Bull. (AAPG)*. **51**, 2246–2259 (1967).

[22] Al Saud, M. Using satellite imageries to study drainage pattern anomalies in Saudi Arabia. *J. Envir Hydro.* ISSN 1058–3912. (2007).

[23] Wray, R. Paleodrainages of the Namoi River Floodplain, New South Wales, Australia: The use of multispectral Landsat imagery to highlight a late quaternary change in fluvial regime. *Austra Geogr.* **40** (1), 29–49 (2009).

[24] Paillou, P. et al. Mapping of a major paleodrainage system in Eastern Libya using orbital imaging Radar: The Kufrah River. *Eart Planet. Scie Lett.* **277**, 327–333. 10.1016/j.epsl.2008.10 (2009).

[25] Samadder, R., Kumar, S. & Gupta, R. Paleodrainages and their potential for artificial groundwater recharge in the western Ganga plains. *J. Hydro.* **400** (1–2), 154–164 (2011).

[26] Paillou, P., Tooth, S. & Lopez, S. The Kufrah paleodrainages system in Libya: A past connection to the Mediterranean Sea? *Compets Rendus. Geos.* **344** (8), 406–414 (2012).

[27] Zhi, C., Cao, W., Wang, Z. & Li, Z. High-Arsenic Groundwater in Paleodrainages of the Lower Yellow River, China: Distribution and Genesis mechanisms. *Water* **13**, 338. 10.3390/w13030338 (2021).

[28] Mulligan, A., Evans, R. & Lizarralde, D. The role of paleodrainages in groundwater/seawater exchange. *J. Hydro.* **335** (3–4), 313–329 (2007).

[29] Rizk, Z., Garamoo, H. & Humaid, R. Impact of a paleochannel on hydrogeochemistry of a quaternary aquifer: Case study from Umm Al Quwain Area, United Arab Emirates. *J. Resea Environ. aEar Scien Issue* (3): 35–46. (2007).

[30] Al Saud, M. Mapping drainage basins of the Kingdom of Saudi Arabia. 20 map sheets (1:500.000). (2024). Technical Study (Under production).

[31] Neal, A. Ground-penetrating radar and its use in sedimentology: principles, problems and progress. *Earth-Sci. Rev.* **66** (3), 261–330 (2004).

[32] Hambly, B. Mapping and characterization of paleodrainages: a comparison of field and remote sensing-based approaches. B.Sc. Dissertation. University of the Sunshine Coast. 59pp. (2015).

[33] Kumar, H. & Rajawat, A. Potential of RISAT-1 SAR data in detecting paleodrainages in parts of the Thar Desert, India. *Curr. Sci.* **113** (10), 1899–1905 (2017).

[34] Khan, Z. & Tewari, R. Paleochannel and paleohydrology of a Middle Siwalik (Pliocene) fluvial system, northern India. *J. Earth Syst. Scie*. **120** (3), 531–543. 10.1007/s12040-011-0083-4 (2011).

[35] Khan, S., Fathy, M. & Abdelazeem, M. Remote sensing and geophysical investigations of Moghra Lake in the Qattara Depression, Western Desert, Egypt. *Geomorphology* **207**, 10–22. 10.1016/j.geomorph.2013.10.023 (2014).

[36] Sajinkumar, K. et al. Migrating rivers, consequent paleochannels: The unlikely partners and hotspots of flooding. *Sci. Total Environ.* **807** (Part 2), 2022150842. 10.1016/j.scitotenv.2021.150842 (2022). 10.1016/j.scitotenv.2021.150842 34627899

[37] Timar, G., Sumegi, P. & Horvath, F. Late quaternary dynamics of the Tisza River: Evidence of climatic and tectonic controls. *Tectonophysics* **410** (1–4), 97–110. 10.1016/j.tecto.2005.06.010 (2005).

[38] Paillou, P., Lopez, S., Marais, E. & Scipal, K. Mapping paleohydrology of the Ephemeral Kuiseb River, Namibia, from Radar Remote sensing. *Water* **12** (5), 1441. 10.3390/w12051441 (2020).

[39] Rossetti, D. Multiple remote sensing techniques as a tool for reconstructing late quaternary drainage in the Amazon lowland. *Earth. Surf. Proc. Land.* **35** (10), 1234–1239. 10.1002/esp.1996 (2010).

[40] Wang, X., Guo, Z., Wu, L., Zhu, C. & He, H. Extraction of paleodrianages information from remote sensing imagery in the east of Chaohu Lake, China. *Front. Environ. Sci.* **6** (1), 75–82 (2012).

[41] Sheng, Y. et al. *Satellite-based Paleo and Recent Lake Changes across the Tibetan Plateau* (AGU, 2017). Abstract id. PP23A-1083.

[42] MPMR - Ministry of Petroleum and Mineral Resources. *Topographic maps (1:50.000) of the Kingdom of Saudi Arabia* (Aerial Survey Department, 1983).

[43] O’Callaghan, F. & Mark, S. The extraction of drainage networks from digital elevation data. *J. Compu Visi Graph Imag. Proce.* **28**, 323–344 (1984).

[44] Upadhyay, R., Kishore, N. & Sharma, M. Delineation and mapping of palaeochannels using remote sensing, geophysical, and sedimentological techniques: A comprehensive approach. *Water Sci.* **35** (1), 100–108. 10.1080/23570008.2021.1941691 (2021).

[45] Owen, R. & Dahlin, T. Inherited drainage-paleodrainages and preferential groundwater flow. *Hydrogeo J.* **18**, 893–903. 10.1007/s10040-010-0588-y (2010).

[46] Crassard, R. et al. Middle Palaeolithic and neolithic occupations around Mundafan palaeolake, Saudi Arabia: Implications for climate change and human dispersals. *PLoS ONE*. **2013** (8), e69665 (2013). 10.1371/journal.pone.0069665 PMC3722113 23894519

[47] Al-Sulaimi, J. & Pitty, A. Origin and depositional model of Wadi Al-Batin and its associated alluvial fan, Saudi Arabia and Kuwait. *Sed Geol.* **97** (1995)), 203–229 (1995).

[48] Pachur, J. & Hoelzmann, P. Late Quaternary palaeoecology and palaeoclimates of the eastern Sahara. *J. Afric. Ear. Sci.* **30**, 929–939 (2000).

[49] Ritambhara, U., Kishore, N. & Sharma, M. Delineation and mapping of paleodrianages using remote sensing, geophysical, and sedimentological techniques: A computer. *Appro Wat Scien*. **35** (1), 100–108. 10.1080/23570008.2021.1941691 (2021).

[50] Albhadili, S., Almallah, I. & Almulla, S. Morphotectonic analysis of Wadi Al-Batin Alluvial Fan, South of Iraq, using remote sensing and GIS techniques. *Ira J. Earth Sci.* **64** (2), 691–707. 10.24996/ijs.2023.64.2.18 (2023).

[51] Bayat, O., Karimzadeh, H. & Karimi, A. Paleo-environment of geomorphic surfaces of an alluvial fan in the eastern Isfahan, Iran, in the light of micromorphology and clay mineralogy. *Arab. J. Geos.* **10**, 91. 10.1007/s12517-017-2848-9 (2017).

[52] Sümeghy, O. & Kiss, T. Morphological and hydrological characteristics of paleo-channels on the alluvial fan of the Maros river, Hungary. *J. Environ. Geograph*. **5** (1–2), 11–19 (2012).

[53] An, F. et al. Drainage geomorphic evolution in response to paleoclimatic changes since 12.8 Ka in the Eastern Kunlun Mountains, NE Qinghai-Tibetan Plateau. *Geomorph* **319**, 117–132. 10.1016/j.geomorph.2018.07.016 (2018).

[54] Haug, K. Modeling Paleo-Flow Events on Alluvial Fans in the Atacama Desert, Chile. Abstracts of International Annual Meetings. ASA-CSSA-SSSA. Nov.1–5, Pittsburgh. (2009).

[55] Sukumar, S. & Sankar, K. Delineation of potential zones for artificial recharge using GIS in Theni district, Tamilnadu, India. *Int. J. Geomat. Geosci.* **1** (3), 639 (2010).

[56] Binnie, S. et al. The origins and implications of paleochannels in hyperarid, tectonically active regions: The northern Atacama Desert, Chile. *Glob. Planet Change* **185**, 103083. 10.1016/j.gloplacha.2019.103083 (2020).

[57] Tan, L. et al. High resolution monsoon precipitation changes on southeastern Tibetan Plateau over the past 2300 years. *Quat. Sci. Rev.* **195**, 122–132. (2018).

[58] Zhou, Y. et al. Response of channel morphology to climate change over the Past 2000 years using vertical boreholes analysis in Lancang River Headwater in Tibetan Plateau. *Water* **14**, 1593. 10.3390/w14101593 (2022).

[59] Selvam, D., Udayaganesan, P. & Ramakrishnan, D. An integrated strategy for the exploration of palaeofluvial placer deposits. *Appl. Geomatics* **13** (2), 165–177. 10.1007/s12518-020-00332-5 (2020).

[60] Thomaz, A., Malabarba, L. R. & Knowles, L. Genomic signatures of paleodrainages in a freshwater fish along the southeastern coast of Brazil: Genetic structure reflects past riverine properties. *Heredity* **119**, 287–294. 10.1038/hdy.2017.46 (2017). 28767104 10.1038/hdy.2017.46 PMC5597787

[61] Muangnoi, S., Chaimanee, N. & Pananont, P. Integrated studies to investigate paleochannel aquifer in Dan Chang District, Suphan Buri Province, Western Thailand. *J. Phys.: Conf. Ser.* **2145** (2022) 012050. (2022). 10.1088/1742-6596/2145/1/012050

[62] White, S. M., Smoak, E., Leier, A. L. & Wilson, A. M. Small muddy paleochannels and implications for submarine groundwater discharge near Charleston, South Carolina, USA. *Geosciences* **13**, 232. 10.3390/geosciences13080232 (2022).

[63] Abdelsalam, G., Robinson, C., El-Baz, F. & Stern. J Applications of orbital imaging radar for geologic studies in arid regions: The Saharan Testimony. *J. Photogramm Eng. Remote Sens.* **66**, 717–726 (2000).

[64] Laben, A. & Brower, V. Process for Enhancing the Spatial Resolution of Multispectral Imagery Using Pansharpening. http://www.google.com/patents/US6011875 (2020).

[65] Craw, D., Kerr, G., Reith, F. & Falconer, D. Pleistocene paleodrainages and placer gold redistribution, western Southland, New Zealand. *NZ J. Geol. Geophys.* **58** (2), 137–153. 10.1080/00288306.2015.1007462 (2015).

[66] Zhou, L. et al. Geological and geochemical characteristics in the paleo-weathering crust sedimentary type REE deposits, western Guizhou, China. *J. Asian Earth Sci.* **73**, 184–198. 10.1016/j.jseaes.2013.04.011 (2013).

[67] Al-Othman, A. Enhancing groundwater recharge in arid region: A case study from Central Saudi Arabia. *Scien Res. Essays* **6** (13), 2757–2762 (2011).

[68] Shaban, A. Dry freshened groundwater conduits: potential sites for groundwater artificial recharge along the continental shelf of Lebanon. American Geophysical Union (AGU). AGU23, Abstract1237000 (2023).

[69] Al Saud, M. Paleochannels: buried conduits storing groundwater and transporting saltwater inland in Saudi Arabia. American Geophysical Union (AGU). AGU23, Abstract1237018 (2023).

